# Galectin-3 in acute myocardial infarction: from molecular mechanisms to clinical translation

**DOI:** 10.3389/fmolb.2025.1730173

**Published:** 2025-12-19

**Authors:** Tingting Liu, Fang Yang

**Affiliations:** Cardiovascular Medicine Department, Yantaishan Hospital, Yantai, China

**Keywords:** acute myocardial infarction, apoptosis, cardiac remodeling, fibrosis, Galectin-3, inflammation, oxidative stress, therapeutic target

## Abstract

**Introduction:**

Acute myocardial infarction is a leading cause of global morbidity and mortality. Galectin-3, a β-galactoside-binding lectin, has been implicated as a key mediator in the pathophysiology following AMI. This review aims to synthesize the evidence on the multifaceted role of galectin-3, spanning from molecular mechanisms to clinical applications.

**Methods:**

A comprehensive literature review was conducted to synthesize current evidence on the molecular functions, biomarker utility, and therapeutic targeting of galectin-3 in AMI. The analysis focused on studies investigating its signaling pathways, clinical correlations, and preclinical interventional models.

**Results:**

Our synthesis demonstrates that galectin-3 acts as a damage-associated molecular pattern that drives critical post-AMI pathologies. Mechanistically, it amplifies inflammation via NF-κB activation and macrophage polarization, promotes fibrosis through synergy with the TGF-β/Smad pathway and fibroblast activation, and regulates cardiomyocyte apoptosis and oxidative/endoplasmic reticulum stress. Clinically, its dynamic expression correlates with infarct size, adverse ventricular remodeling, and poor outcomes. As a biomarker, elevated circulating galectin-3 predicts major adverse cardiovascular events, heart failure, and mortality, improving risk stratification in multi-marker panels. Serial measurements indicate treatment response, with declining levels post-PCI or statin therapy associated with improved prognosis. Therapeutically, both genetic ablation and pharmacological inhibition of galectin-3 attenuate inflammation, fibrosis, and cardiac dysfunction in preclinical models.

**Discussion:**

Galectin-3 occupies a critical position at the intersection of AMI pathogenesis, diagnosis, and therapy. Targeting the galectin-3 pathway represents a promising therapeutic strategy to improve post-AMI outcomes, although its clinical translation requires further investigation. This review underscores the potential of integrating galectin-3 assessment and inhibition into future AMI management strategies.

## 1 Introduction: epidemiology and pathophysiology of acute myocardial infarction

Acute myocardial infarction (AMI) remains a leading cause of global cardiovascular death, with 800000 annual cases and persistent 30-day mortality rates of 6%–8% despite reperfusion advances (Bhatt et al., 2022). Critically, heart failure (HF) complicates 25.5% of AMI cases, doubling 1-year mortality risk (HR = 2.1; 95% CI 1.72–2.34) and accounting for 40% of 30-day readmissions (Goodwin et al., 2023; Harrington et al., 2022). The economic burden exceeds $11 billion/year in the US alone, underscoring unmet clinical needs (Goodwin et al., 2023).

Ischemia-reperfusion (I-R) injury triggers a maladaptive inflammatory cascade: Neutrophil infiltration releases MMP-9 and ROS within 6 h, expanding infarct size by 40% (Timmers et al., 2012). TRPM2 channel activation induces mitochondrial Ca^2+^ overload, increasing apoptosis 3.5-fold (Huang et al., 2024). Sustained inflammation drives collagen deposition, reducing LVEF by >10% in 25% of revascularized patients (Westman et al., 2016; Algoet et al., 2023).

**FIGURE 1:**
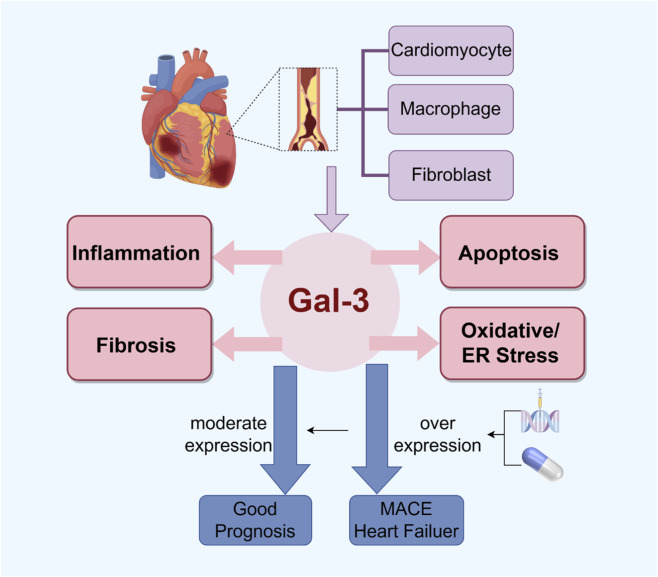
The role of Gal-3 in AMI. Top: Following AMI, Gal-3 is released from necrotic cardiomyocytes and secreted by activated macrophages and fibroblasts. Middle: Gal-3 drives key pathological processes including inflammation, fibrosis, apoptosis, and oxidative/ER stress. Bottom: Clinically, over expression of Gal-3 serves as a prognostic biomarker for MACE and heart failure. Therapeutic strategies include genetic knockdown, pharmacological inhibitors and repurposed drugs, which attenuate Gal-3-mediated pathology and improve cardiac outcomes. Abbreviations: Gal-3, Galectin-3; AMI, Acute Myocardial Infarction; ER, Endoplasmic Reticulum; MACE, Major Adverse Cardiovascular Events.

Although primary percutaneous coronary intervention (PCI) reduces infarct size by 30%–50% and restores coronary blood flow, it does not effectively modulate the fibrotic response that underpins adverse ventricular remodeling (Bhatt et al., 2022). This limitation is compounded by the insufficient performance of conventional biomarkers in predicting post-infarction fibrosis and clinical outcomes. In this context, galectin-3 (Gal-3)—a macrophage-derived β-galactoside-binding lectin—has emerged as a central regulator of key AMI pathologies, including inflammation, fibroblast activation, and collagen deposition, offering promise as both a prognostic biomarker and a therapeutic target (Figure 1).

## 2 Biological characteristics of Galectin-3

Gal-3, a unique 30–35 kDa protein, stands as the sole chimera-type member within the evolutionarily conserved galectin family of β-galactoside-binding lectins. Its distinctive molecular architecture underpins its remarkable functional versatility. Structurally, Gal-3 comprises an N-terminal domain rich in proline, glycine, and tyrosine residues, which facilitates oligomerization, and a single C-terminal carbohydrate recognition domain (CRD) responsible for binding β-galactoside-containing glycoconjugates (Sciacchitano et al., 2018; Soares et al., 2021). This N-terminal domain harbors sites susceptible to serine phosphorylation and cleavage by matrix metalloproteinases (MMPs), adding layers of post-translational regulation. The CRD contains the conserved carbohydrate-binding motifs and a distinctive NWGR anti-death motif implicated in some intracellular functions (Díaz-Alvarez and Ortega, 2017). Gal-3 exhibits dynamic subcellular localization. Within the cytoplasm and nucleus, it participates in critical processes such as cell cycle regulation, proliferation, apoptosis inhibition, RNA splicing, and mRNA stability (Sciacchitano et al., 2018; Li et al., 2022; Seropian et al., 2023). Upon secretion, which occurs via non-classical pathways independent of the Golgi apparatus, Gal-3 functions extracellularly. Here, its ability to oligomerize via the N-terminal domain allows it to cross-link glycoproteins on cell surfaces or within the extracellular matrix (ECM), mediating cell-cell and cell-ECM adhesion, transmembrane signaling, and lattice formation that modulates receptor dynamics (Sciacchitano et al., 2018; Soares et al., 2021).

The biological functions of Galectin-3 are exceptionally pleiotropic, underpinning its involvement in a vast spectrum of physiological and pathological processes across nearly all organ systems. In cardiovascular diseases, it is a well-established mediator of cardiac fibrosis, adverse remodeling, and heart failure progression, serving as both a key pathogenic driver and a valuable prognostic biomarker (Zhong et al., 2019; Procyk et al., 2023; Zhang et al., 2024). Its role extends to arrhythmogenesis, particularly in promoting atrial fibrillation by contributing to atrial structural and electrical remodeling (Procyk et al., 2023). Beyond the heart, Gal-3 is implicated in neurological disorders, where it regulates neuroinflammation, microglial activation, and contributes to the pathogenesis of conditions like Alzheimer’s disease and stroke (Soares et al., 2021; Qiu et al., 2024). In metabolic diseases, Gal-3 is linked to insulin resistance, diabetic complications, and the progression of atherosclerosis (Lu et al., 2017; Jimenez-Gonzalez et al., 2024). Its canonical role in cancer is profound, where it promotes tumor cell proliferation, angiogenesis, metastasis, and immune evasion (Sciacchitano et al., 2018). Furthermore, Gal-3 is a critical modulator of innate and adaptive immune responses, influencing macrophage polarization, neutrophil function, and T-cell homeostasis, which links it to autoimmune diseases, infectious diseases, and chronic inflammatory conditions (Díaz-Alvarez and Ortega, 2017; Kovacevic et al., 2018; Souza et al., 2017). It also plays significant roles in renal fibrosis, acute kidney injury, liver fibrosis, and ocular pathologies, demonstrating its ubiquitous influence (Li et al., 2022; Horiuchi et al., 2023). This remarkable functional diversity stems from its ability to interact with a wide array of glycoprotein ligands, its dynamic localization, and its context-dependent actions, making it a molecule of intense interest for diagnostic and therapeutic targeting across myriad human diseases.

## 3 Dynamic expression of Galectin-3 in AMI

### 3.1 Spatiotemporal expression dynamics of Galectin-3 in AMI

Gal-3 exhibits a highly dynamic temporal expression profile following AMI, closely mirroring the phases of injury, inflammation, and subsequent remodeling. Circulating levels of Gal-3 rise remarkably early after the ischemic insult. Clinical studies consistently demonstrate a significant increase in serum Gal-3 detectable within hours of AMI onset. Bivona et al. (2016) reported a rapid and substantial elevation in plasma Gal-3 levels as early as 4–6 h post-admission in STEMI patients, peaking significantly within the first 24–72 h compared to healthy controls and patients with stable angina. This early surge correlates strongly with established markers of myocardial necrosis; Alturfan et al. (2014) found a positive correlation between peak serum Gal-3 levels within the first 48 h and peak cardiac troponin I (cTnI) levels, as well as creatine kinase-MB (CK-MB) activity, suggesting its release is intimately linked to the extent of cardiomyocyte death. Histological evidence from animal models and human autopsy specimens confirms this rapid myocardial upregulation. Hashmi and Al-Salam (2015) observed intense Gal-3 immunostaining within the infarct zone of human hearts obtained within just 6 h of fatal AMI, primarily localized to infiltrating leukocytes and damaged cardiomyocytes at this very early stage. Sharma et al. (2017), in a detailed temporal study using a rat MI model, quantified myocardial Gal-3 mRNA and protein, showing a steep rise starting at 6 h, peaking dramatically between day 1 and day 3 post-MI, coinciding with the peak of inflammatory cell infiltration. While serum levels typically decline from their peak after the first week (days 4–7), they often remain persistently elevated above baseline for weeks to months (Sharma et al., 2017; Meijers et al., 2015). This sustained elevation is clinically significant, as it correlates with ongoing adverse left ventricular remodeling, progressive fibrosis, and the development of heart failure. Recent data also suggests potential differences in kinetics between STEMI and NSTEMI, with STEMI patients potentially showing a more pronounced early rise (Misirlioglu et al., 2025). The persistence of elevated Gal-3, both in the myocardium and circulation, serves as a biomarker reflecting the chronicity of the inflammatory and fibrotic processes driving post-infarct remodeling (Meijers et al., 2015; Frunza et al., 2016).

Spatially, the expression of Gal-3 post-AMI is highly compartmentalized and cell-type specific, evolving dynamically within the infarcted heart. The highest concentration of Gal-3 protein is found within the infarct core and the critically important border zone during the acute and subacute phases (days 1–7). Within these regions, Gal-3 is expressed by multiple cell types, but its cellular sources shift over time. In the very early hours (0–24 h), damaged and necrotic cardiomyocytes themselves contribute to Gal-3 release as a Damage-Associated Molecular Pattern (DAMP) (Hashmi and Al-Salam, 2015). However, the predominant and most sustained source rapidly becomes the infiltrating inflammatory cells. Sharma et al. (2017) demonstrated that Gal-3 expression colocalized intensely with CD68^+^ macrophages and, to a lesser extent, neutrophils within the infarct and border zones, peaking around day 3–5 post-MI in rats. This macrophage-derived Gal-3 is a major driver of the local inflammatory response and subsequent fibrotic cascade. As the acute inflammation subsides and the reparative phase progresses (days 7 onwards), activated cardiac fibroblasts and myofibroblasts become significant producers of Gal-3 within the evolving scar tissue and the remodeling myocardium (Sharma et al., 2017; Frunza et al., 2016). Frunza et al. (2016) highlighted the strong association between fibroblast-derived Gal-3 expression and collagen deposition during the pressure-overload induced remodeling process, a finding highly relevant to post-MI fibrosis. Endothelial cells within the infarct-related vasculature also express Gal-3, potentially contributing to leukocyte recruitment and endothelial activation (Meijers et al., 2015). Importantly, the spatial gradient of Gal-3 expression is significant: levels are highest in the dense infarct core, intermediate in the border zone where viable and dying cardiomyocytes intermingle with inflammatory cells and activated fibroblasts, and substantially lower in the remote, non-infarcted myocardium, although remote area expression may increase later during chronic remodeling (Sharma et al., 2017). The spatial distribution directly impacts function; high local concentrations in the infarct and border zone facilitate Gal-3 oligomerization, enhancing its pro-inflammatory and pro-fibrotic signaling potency. Circulating Gal-3 levels largely reflect the integrated output of these cellular sources, predominantly activated macrophages and fibroblasts, within the injured myocardium (Sharma et al., 2017; Meijers et al., 2015).

### 3.2 Regulatory mechanisms of Galectin-3 expression in AMI

The robust upregulation of Gal-3 observed in AMI is orchestrated by a complex interplay of transcriptional, cellular, and microenvironmental factors, primarily driven by the ischemic and inflammatory milieu. At the transcriptional level, activation of the nuclear factor-kappa B (NF-κB) signaling pathway serves as a master regulator of *LGALS3* gene expression. I/R injury, hypoxia/reoxygenation (H/R) stress, and the release of DAMPs activate Toll-like receptors (TLRs), particularly TLR4, culminating in the phosphorylation of IκBα, nuclear translocation of the p65 subunit, and binding to κB sites within the *LGALS3* promoter (Suthahar et al., 2018; Mosleh et al., 2018; Li et al., 2023). This establishes a potent positive feedback loop, as Gal-3 itself acts as a DAMP, further amplifying NF-κB activation and its own synthesis. Hypoxia-inducible factors (HIFs), stabilized under low oxygen tension, also contribute to Gal-3 transcriptional induction in cardiomyocytes and infiltrating cells within the infarct zone (Yalta et al., 2020). Furthermore, pro-inflammatory cytokines like tumor necrosis factor-alpha (TNF-α) and interleukin-1 beta (IL-1β), abundantly produced early after AMI, directly stimulate Gal-3 expression in various cell types, reinforcing the inflammatory response (Suthahar et al., 2018).

The cellular source dynamics significantly contribute to the regulation of Gal-3 levels within the infarcted heart. While damaged cardiomyocytes release stored Gal-3 acutely, the sustained and predominant production rapidly shifts to infiltrating inflammatory cells, especially activated macrophages. The recruitment and activation of monocytes/macrophages are themselves triggered by the initial ischemic insult and early chemokine release. Once present, these cells become major producers of Gal-3, significantly amplifying its local concentration (Li et al., 2023). Genetic deletion studies in mice demonstrate that macrophage-derived Gal-3 critically influences the kinetics and phenotype of the macrophage infiltrate itself, altering the temporal evolution of inflammation and healing, thereby impacting subsequent remodeling (Li et al., 2023). As the acute inflammatory phase transitions into the proliferative phase, activated cardiac fibroblasts and differentiating myofibroblasts become key contributors to Gal-3 expression, driven by factors like transforming growth factor-beta (TGF-β) and mechanical stress associated with ventricular wall strain, linking Gal-3 directly to the fibrotic response (Suthahar et al., 2018; Yalta et al., 2020).

Pharmacological interventions provide evidence for modulating Gal-3 expression as a therapeutic strategy and further illuminate regulatory pathways. Studies demonstrate that drugs like Ticagrelor, a P2Y12 receptor antagonist used in AMI, can attenuate myocardial I/R injury partly through downregulating Gal-3 expression in the infarct area. This effect is associated with reduced oxidative stress and inflammation, suggesting Ticagrelor may interfere with upstream signals (e.g., platelet activation, inflammation) that drive Gal-3 transcription (Liu et al., 2018). Direct targeting of Gal-3 using specific inhibitors (e.g., Modified Citrus Pectin - MCP, small molecule inhibitors like TD139) not only blocks its function but can also influence its expression levels in some contexts, potentially by disrupting autocrine/paracrine loops or feedback mechanisms (Mosleh et al., 2018; Mo et al., 2019). For instance, Gal-3 blockade has been shown to mitigate inflammation in the infarct region, which could secondarily reduce the stimuli (cytokines, DAMPs) driving further Gal-3 production (Mosleh et al., 2018). The timing of intervention is crucial, as early inhibition might target the initial DAMP-driven inflammation, while later inhibition could more effectively dampen the fibrogenic actions driven by sustained fibroblast expression (Suthahar et al., 2018; Yalta et al., 2020).

## 4 The molecular mechanisms of Galectin-3 in AMI

### 4.1 Inflammation and fibrosis: core pathological mechanisms orchestrated by Galectin-3 in AMI

**FIGURE 2:**
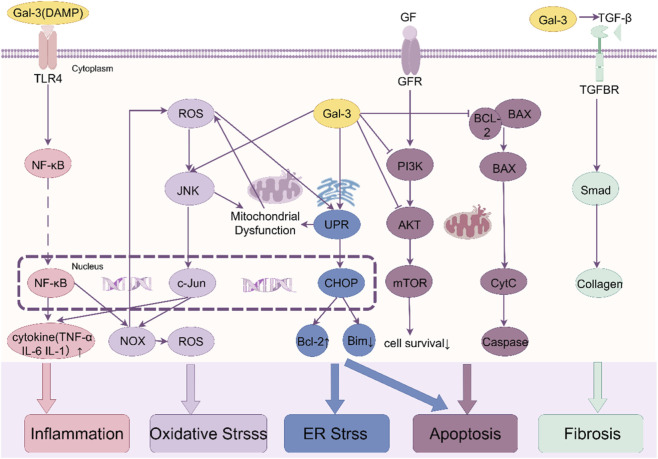
Molecular Mechanisms of Gal-3 in AMI Pathophysiology. Galectin-3 orchestrates multiple pathological processes in AMI through key signaling pathways. Inflammation: Gal-3 acts as a DAMP, binding TLR4 and activating NF-κB, leading to cytokine release. Fibrosis: Gal-3 synergizes with TGF-β/Smad signaling, promoting fibroblast activation and collagen deposition. Apoptosis: Gal-3 inhibits PI3K/Akt and interacts with Bcl-2, promoting mitochondrial dysfunction and caspase activation. Oxidative and ER Stress: Gal-3 enhances ROS production and ER stress via JNK and PERK-CHOP pathways. Abbreviations: AMI, Acute Myocardial Infarction; Gal-3, Galectin-3; DAMP, Damage-Associated Molecular Pattern; TLR4, Toll-like receptor 4; TGF-β, transforming growth factor-beta; ROS, reactive oxygen species; ER, endoplasmic reticulum.

Gal-3 is a pivotal amplifier of the detrimental inflammatory cascade that ensues following AMI. Released as a DAMP from necrotic cardiomyocytes, Gal-3 binds directly to pattern recognition receptors, notably Toll-like receptor 4 (TLR4), on infiltrating immune cells (macrophages, neutrophils) and resident cardiac cells (Zhang et al., 2015; Yang et al., 2016; Burguillos et al., 2015). This binding triggers robust activation of the NF-κB signaling pathway, characterized by IκBα phosphorylation, p65 nuclear translocation, and subsequent transcription of potent pro-inflammatory cytokines, including TNF-α, IL-1β, and interleukin-6 (IL-6) (Westman et al., 2016; Jimenez-Gonzalez et al., 2024; Wang et al., 2023) (Figure 2). Gal-3 thus establishes a powerful positive feedback loop: initial inflammation promotes further Gal-3 release and synthesis, which in turn sustains and amplifies the inflammatory response. Gal-3 also promotes neutrophil infiltration and activation, exacerbating injury through reactive oxygen species (ROS) generation and protease release (Daseke et al., 2021). Genetic deletion or pharmacological inhibition of Gal-3 consistently demonstrates attenuated myocardial inflammation, reduced inflammatory cell infiltration, and lower cytokine levels in experimental AMI models, confirming its central pro-inflammatory role (Yu et al., 2013).

Gal-3 is a master regulator of cardiac fibrosis, a hallmark of adverse ventricular remodeling post-AMI that leads to diastolic and systolic dysfunction and heart failure progression. It exerts potent pro-fibrotic effects through direct actions on cardiac fibroblasts and indirect mechanisms. Gal-3 directly stimulates fibroblast proliferation, activation, and differentiation into collagen-secreting myofibroblasts, identified by increased alpha-smooth muscle actin (α-SMA) expression (Zhong et al., 2019; Yu et al., 2013; Besler et al., 2017). It potently enhances the synthesis and deposition of ECM components, particularly fibrillar collagens type I and III. Crucially, Gal-3 acts synergistically with and amplifies signaling through the canonical transforming growth factor-beta 1 (TGF-β1)/Smad pathway, a primary driver of fibrosis (Wang et al., 2023; Wan et al., 2022; Prabhu and Frangogiannis, 2016). Gal-3 can stabilize TGF-β receptors and enhance Smad2/3 phosphorylation, maximizing the fibrotic output of TGF-β1 (Chen et al., 2022) (Figure 2). Additionally, Gal-3 critically orchestrates macrophage polarization towards a pro-inflammatory and pro-fibrotic phenotype (a process detailed in Section 4.5), thereby amplifying tissue damage and fibrogenic signaling (Martínez-Martínez et al., 2015; Ma et al., 2018; Yap et al., 2023). Gal-3 also contributes to fibrosis by modulating oxidative stress and endoplasmic reticulum (ER) stress pathways, which are interconnected with inflammation and fibroblast activation (Jimenez-Gonzalez et al., 2024).

Targeting Gal-3 presents a promising strategy to disrupt the intertwined inflammation-fibrosis axis in AMI. Both genetic deletion and pharmacological inhibition of Gal-3 confer significant cardio-protection in preclinical models, effectively attenuating inflammation, fibrosis, adverse remodeling, and cardiac dysfunction (Jimenez-Gonzalez et al., 2024; Wang et al., 2023; Yu et al., 2013; Martínez-Martínez et al., 2015; Li et al., 2023). The efficacy of Gal-3 blockade highlights its potential as a therapeutic target to improve long-term outcomes after AMI by promoting more favorable wound healing and reducing the risk of heart failure.

### 4.2 Regulation of cardiomyocyte apoptosis and survival by Galectin-3 in AMI

Gal-3 exerts complex and context-dependent effects on cardiomyocyte fate during AMI, predominantly promoting apoptosis under the severe stress of ischemia/reperfusion (I/R) injury. A key mechanism involves Gal-3’s inhibition of pro-survival signaling pathways. Gal-3 suppresses the activation of the PI3K/Akt pathway, a critical cascade for cardiomyocyte survival. By interfering with Akt phosphorylation and activation, Gal-3 diminishes the downstream anti-apoptotic signals, thereby sensitizing cardiomyocytes to apoptotic stimuli such as hypoxia and oxidative stress (Sun et al., 2021; Luo et al., 2023). This inhibition of Akt signaling is particularly pronounced in pathological conditions like hyperglycemia and hyperlipidemia, which often coexist with AMI (Sun et al., 2021). Furthermore, Gal-3 directly interacts with the anti-apoptotic protein Bcl-2. This interaction compromises Bcl-2’s ability to sequester pro-apoptotic proteins like Bax, leading to increased mitochondrial outer membrane permeabilization (MOMP), cytochrome c release, and activation of the caspase cascade, culminating in apoptotic cell death (Mo et al., 2019; Zhang et al., 2020). Gal-3 also contributes to the activation of pro-apoptotic pathways, including the JNK/c-Jun pathway, further tipping the balance towards cell death (Luo et al., 2023). Additionally, Gal-3 exacerbates cardiomyocyte apoptosis under metabolic comorbidities by amplifying endoplasmic reticulum (ER) stress, leading to CHOP-mediated apoptotic pathways (detailed in Section 4.3) (Jimenez-Gonzalez et al., 2024). Collectively, these mechanisms position Gal-3 as a significant contributor to the loss of viable myocardium in the infarct and border zones (Figure 2).

Paradoxically, while Gal-3 promotes apoptosis in the stressed cardiomyocytes of the infarcted heart, evidence suggests it can exert anti-apoptotic effects in certain cellular contexts or under different stress conditions (e.g., some cancer cells or non-ischemic settings) (Funasaka et al., 2014). However, the net effect of endogenous Gal-3 in the AMI milieu appears overwhelmingly pro-apoptotic. Genetic and pharmacological interventions targeting Gal-3 provide compelling support for its detrimental role in cardiomyocyte survival post-AMI. Knockdown or knockout of Gal-3 significantly reduces cardiomyocyte apoptosis in experimental models of cardiac I/R injury. Zhang et al. (2020) demonstrated that knocking down Gal-3 expression protected cardiomyocytes against I/R-induced apoptosis, an effect associated with restored Bcl-2 function and reduced caspase-3 activation. Similarly, pharmacological inhibition of Gal-3 using agents like MCP or specific small molecules (e.g., TD139 analogs) consistently demonstrates a reduction in apoptotic cardiomyocyte death following I/R injury or AMI (Mo et al., 2019; Yu et al., 2013). These interventions often correlate with improved preservation of myocardial tissue and cardiac function. The detrimental pro-apoptotic role of Gal-3 is further accentuated in the presence of metabolic stress, such as obesity, where its synergy with ER stress pathways creates a particularly hostile environment for cardiomyocyte survival. Therefore, inhibiting Gal-3 represents a promising strategy to limit cardiomyocyte loss, a critical determinant of infarct size and subsequent ventricular remodeling.

### 4.3 Oxidative stress and endoplasmic reticulum stress: amplifying injury through Galectin-3

Gal-3 significantly exacerbates oxidative stress in the post-AMI myocardium through multiple intertwined mechanisms. Building upon its activation of the TLR4/NF-κB pathway, Gal-3 upregulates NADPH oxidase (NOX) expression and promotes ROS generation in cardiomyocytes and inflammatory cells (Al-Salam and Hashmi, 2018; Hassan et al., 2023). Concurrently, Gal-3 inhibits endogenous antioxidant defenses, including superoxide dismutase (SOD) and glutathione peroxidase (GPx), creating a redox imbalance (Fulton et al., 2019). This oxidative burst activates stress kinases: Gal-3 triggers the JNK/c-Jun pathway, which further amplifies ROS production and promotes mitochondrial dysfunction, leading to cytochrome c release and caspase activation (Zhang et al., 2018; Al-Salam et al., 2022). The ROS-JNK-Gal-3 axis establishes a vicious cycle, as oxidative stress itself enhances Gal-3 expression via NF-κB (Jimenez-Gonzalez et al., 2024). Pharmacological inhibition of Gal-3 (e.g., TD139) reduces JNK phosphorylation and suppresses ROS overproduction, confirming its pivotal role in redox dysregulation (Xia et al., 2024) (Figure 2).

Gal-3 critically modulates ER stress pathways, particularly in metabolically compromised hearts. During AMI, I-R disrupts ER proteostasis, triggering the unfolded protein response (UPR). Gal-3 binds to and stabilizes key UPR sensors (e.g., PERK), prolonging the activation of the PERK-eIF2α-CHOP arm (Jimenez-Gonzalez et al., 2024; Al-Salam and Hashmi, 2018). This sustains CHOP expression, which suppresses anti-apoptotic Bcl-2, upregulates pro-apoptotic Bim, and drives caspase-12-mediated apoptosis (Al-Salam et al., 2022) (Figure 2). Notably, in obesity-related AMI models, Gal-3 synergizes with metabolic stressors (e.g., lipotoxicity) to amplify ER stress, leading to severe cardiomyocyte loss and fibrosis (Jimenez-Gonzalez et al., 2024). Gal-3 also bridges ER stress with inflammation by facilitating NF-κB nuclear translocation and TNF-α secretion (Hassan et al., 2023). Inhibition strategies (e.g.,,MCP) disrupt Gal-3–UPR interactions, reducing CHOP expression and improving cell survival (Fulton et al., 2019), highlighting its potential as a therapeutic node for dual stress mitigation.

### 4.4 Crosstalk with other signaling pathways: expanding Galectin-3’s influence in AMI pathogenesis

Gal-3 exhibits significant crosstalk with the Wnt/β-catenin signaling pathway, amplifying pro-fibrotic signaling critical for adverse cardiac remodeling post-AMI. Gal-3 enhances canonical Wnt signaling by stabilizing β-catenin, the key transcriptional effector. Mechanistically, Gal-3 inhibits glycogen synthase kinase-3β (GSK-3β), the kinase responsible for phosphorylating β-catenin and targeting it for proteasomal degradation (Liu et al., 2023; Xu et al., 2022). This stabilization leads to increased nuclear translocation of β-catenin, where it complexes with TCF/LEF transcription factors to drive the expression of pro-fibrotic genes (e.g., fibronectin, collagen types I and III, MMPs) and genes promoting fibroblast proliferation and activation (Liu et al., 2023; Kim et al., 2021). Conversely, Wnt ligands can stimulate Gal-3 expression, creating a positive feedback loop that sustains fibrotic activity within the infarct and border zones (Xu et al., 2022; Al-Dalahmah et al., 2020). This synergistic interaction positions the Gal-3/Wnt/β-catenin axis as a potent driver of pathological extracellular matrix deposition and ventricular stiffening. Inhibition of Gal-3 disrupts this crosstalk, reducing β-catenin nuclear accumulation and downstream fibrogenic gene expression, thereby mitigating cardiac fibrosis in experimental models (Liu et al., 2023).

Gal-3 also modulates the Notch signaling pathway, which plays complex roles in cardiac repair, angiogenesis, and potentially stem cell-mediated regeneration following AMI. While direct mechanistic evidence specifically in AMI is evolving, studies in other contexts indicate Gal-3 can influence Notch ligand-receptor interactions or downstream signaling events. Gal-3 may bind to glycans on Notch receptors or ligands (e.g., Jagged1, DLL4), potentially altering their availability or activity at the cell surface (Cai and Dai, 2025). This interaction could impact critical Notch-mediated processes in the post-infarct heart, such as regulating the balance between cardiomyocyte survival/apoptosis, modulating endothelial cell function and angiogenesis within the peri-infarct region, and influencing the behavior and differentiation potential of cardiac progenitor cells or other reparative cell populations (Cai and Dai, 2025). Al-Dalahmah et al. (2020) demonstrated Gal-3’s capacity to diminish Wnt signaling in neural stem cells, highlighting its broader role in modulating developmental/growth pathways relevant to tissue repair. The interplay between Gal-3 and Notch likely influences the efficiency and quality of cardiac healing and scar formation, representing an emerging area for investigation to understand its contribution to regeneration versus maladaptive remodeling after AMI (Kim et al., 2021; Cai and Dai, 2025).

### 4.5 Immune modulation and macrophage polarization: Galectin-3 as a master regulator

Gal-3 plays a pivotal role in shaping the immune response within the infarcted myocardium, primarily by critically regulating macrophage recruitment, activation, and polarization. Released as a DAMP and expressed by activated macrophages themselves, Gal-3 acts as a potent chemoattractant and activator, amplifying the infiltration of monocytes/macrophages into the ischemic zone (Mosleh et al., 2018; Li et al., 2023). Once present, Gal-3 profoundly influences macrophage phenotype. Substantial evidence indicates that Gal-3 drives macrophages towards a pro-inflammatory (M1-like) state early post-AMI, characterized by enhanced production of cytokines like TNF-α, IL-1β, and IL-6, thereby exacerbating tissue injury (Yap et al., 2023; Li et al., 2023). As the inflammatory phase progresses, Gal-3 promotes a transition towards or sustains a pro-fibrotic macrophage phenotype (often associated with M2 markers like CD206 or Arg1, but functionally distinct and detrimental in this context). These Gal-3-polarized macrophages secrete high levels of TGF-β1 and other fibrogenic factors, directly fueling cardiac fibroblast activation and collagen deposition (Wang et al., 2024; Li et al., 2023). Recent research reveals a key mechanism: Gal-3 interacts with Triggering Receptor Expressed on Myeloid Cells-2 (TREM2) on macrophages. This interaction impairs TREM2-mediated phagocytic clearance of debris and apoptotic cells (efferocytosis) and actively reprograms macrophages towards a pathogenic, immunosuppressive state, akin to tumor-associated macrophages (TAMs), which are highly pro-fibrotic and hinder resolution (Wang et al., 2024). Genetic deletion of Gal-3 significantly alters the dynamics of macrophage infiltration post-MI, reducing the number of pro-inflammatory/pro-fibrotic macrophages, delaying their phenotypic shift, and ultimately leading to attenuated inflammation, reduced fibrosis, and improved cardiac function and remodeling (Dick et al., 2019; Li et al., 2023). This highlights Gal-3 as a central orchestrator of macrophage-mediated pathology in AMI.

### 4.6 Metabolic regulation: Galectin-3 at the intersection of metabolism and cardiac injury

Gal-3 plays a significant role in metabolic dysregulation within the context of AMI, particularly influencing and being influenced by lipid metabolism, which impacts cardiac remodeling outcomes. Clinical evidence demonstrates a robust inverse correlation between circulating levels of marine-derived omega-3 polyunsaturated fatty acids (n-3 PUFAs), such as eicosapentaenoic acid (EPA) and docosahexaenoic acid (DHA), and serum Gal-3 concentrations (Laake et al., 2017). Mechanistically, n-3 PUFAs may exert cardioprotective effects partly by suppressing Gal-3 expression or activity. Proposed pathways include activation of peroxisome proliferator-activated receptor gamma (PPARγ), which can inhibit Gal-3 transcription, and modulation of inflammatory signaling cascades upstream of Gal-3 induction (e.g., NF-κB) (Laake et al., 2017; Kwong et al., 2019). Conversely, elevated Gal-3 levels, commonly observed in metabolic syndrome, obesity, and diabetes–frequent comorbidities in AMI patients–contribute to insulin resistance and impaired glucose metabolism. Gal-3 contributes to insulin resistance, in part by inhibiting the PI3K/Akt survival signaling axis (as also implicated in apoptosis, Section 4.2), exacerbating metabolic stress (Sun et al., 2021). This metabolic stress, characterized by lipotoxicity, glucotoxicity, and mitochondrial dysfunction, synergizes with Gal-3 to amplify ER stress, oxidative stress, inflammation, and ultimately, adverse cardiac remodeling and dysfunction post-AMI (Jimenez-Gonzalez et al., 2024). This bidirectional relationship positions Gal-3 as a crucial mediator linking metabolic dyshomeostasis to worse outcomes after AMI, suggesting that interventions targeting n-3 PUFA status or Gal-3 activity could be therapeutically beneficial (Kwong et al., 2019).

### 4.7 Interaction with lipid rafts: Galectin-3-mediated signaling transduction and membrane function regulation

Emerging evidence suggests that Galectin-3’s functions are intricately linked to its association with cholesterol-rich membrane microdomains known as lipid rafts. Galectin-3 can localize to and modulate the composition and stability of lipid rafts, thereby influencing the spatial organization and signaling efficiency of receptors concentrated within these domains, such as Toll-like receptors and growth factor receptors (Liu et al., 2025). This compartmentalization amplifies downstream pro-inflammatory and pro-fibrotic signaling cascades. The integrity of lipid rafts is essential for Gal-3-mediated processes such as macrophage polarization and endothelial cell activation (Matthews et al., 2023). Consequently, therapeutic strategies targeting lipid raft integrity—such as statins and methyl-β-cyclodextrin may disrupt Gal-3’s signaling platform. For instance, cyclodextrin-mediated raft disruption enhances endothelial nitric oxide production, potentially counteracting Gal-3-driven endothelial dysfunction and inflammation (Riitano et al., 2023; Warda et al., 2025). This intersection highlights lipid rafts as a potential therapeutic node to modulate Gal-3-dependent pathways in AMI.

## 5 Clinical value of Galectin-3 as a biomarker

### 5.1 Prognostic prediction: Galectin-3 as a robust indicator of adverse outcomes

Elevated circulating Gal-3 levels, particularly measured early (within the first 24–72 h) after AMI, are consistently and independently associated with a significantly increased risk of major adverse cardiovascular events (MACE) and mortality in both the short and long term. Numerous large-scale clinical studies and meta-analyses have solidified this relationship. Patients with Gal-3 levels above established thresholds (e.g., >17.8 ng/mL, >25.9 ng/mL, or above the median/75th percentile of the study population) exhibit substantially higher rates of death, recurrent myocardial infarction, hospitalization for HF, and the development of post-infarction HF compared to those with lower levels (Asleh et al., 2019; Tian et al., 2020; Kokturk et al., 2023). For instance, in a large population-based study, AMI patients with Gal-3 levels >25.9 ng/mL had a 3-fold higher risk of all-cause mortality over 5 years compared to those with levels <17.8 ng/mL (Asleh et al., 2019). A comprehensive meta-analysis confirmed that elevated Gal-3 post-AMI was a significant predictor of all-cause mortality (HR 1.78), cardiovascular mortality (HR 1.92), and HF hospitalization (HR 1.54) (Tian et al., 2020). This predictive power holds true across different AMI subtypes, including both ST-elevation MI (STEMI) and non-ST-elevation MI (NSTEMI), although the optimal prognostic thresholds may vary slightly (Kokturk et al., 2023; Obeid et al., 2020) (Table 1).

**TABLE 1:** Summary of major clinical studies evaluating Galectin-3 for predicting outcomes in AMI patients.

Study (Year)	Population	Key findings	Threshold (ng/mL)	Outcomes predicted
Asleh et al. (2019)	Population-based	Gal-3 >25.9 ng/mL associated with 3-fold higher 5-year mortality	>25.9	All-cause mortality
Tian et al. (2020)	Meta-analysis	HR 1.78 for all-cause mortality; HR 1.54 for HF hospitalization	Various	Mortality, HF
Kokturk et al. (2023)	STEMI post-PCI	High Gal-3 predicts long-term MACE and mortality	>17.8	MACE, mortality
Obeid et al. (2020)	ACS patients	Gal-3 independently predicts 1-year mortality and HF	>17.8	Mortality, HF

Abbreviations: Gal-3, Galectin-3; HF, heart failure; PCI, percutaneous coronary intervention; MACE, Major Adverse Cardiovascular Events. STEMI, ST-Elevation Myocardial Infarction.

The prognostic value of Gal-3 stems from its direct reflection of key pathological processes driving adverse outcomes: inflammation, fibrosis, and adverse ventricular remodeling. Gal-3 levels measured early post-AMI correlate strongly with markers of infarct size (e.g., peak troponin, CK-MB), impaired left ventricular ejection fraction (LVEF) at discharge, and, crucially, with the severity and progression of adverse LV remodeling (Di Tano et al., 2017; van der Velde et al., 2016; Perea et al., 2016). Patients with high Gal-3 levels are more likely to develop progressive LV dilation, wall thinning, and systolic dysfunction. Advanced imaging techniques like cardiac magnetic resonance (CMR) confirm that elevated Gal-3 correlates with larger infarct size, greater microvascular obstruction, and increased extracellular volume (ECV) fraction–a marker of diffuse fibrosis–in both infarcted and remote myocardium, providing a structural basis for the increased risk (Perea et al., 2016; Dong et al., 2018). Importantly, Gal-3 provides incremental prognostic information beyond traditional risk factors (age, comorbidities), clinical risk scores, and established biomarkers such as B-type natriuretic peptide (BNP) or N-terminal pro-BNP (NT-proBNP) (van der Velde et al., 2016; Ghorbani et al., 2018; Vucic et al., 2023). Serial measurements further enhance its utility; persistently high or rising Gal-3 levels during follow-up signify ongoing adverse remodeling and portend a worse prognosis compared to patients whose levels decline (Sharma et al., 2017; Ghorbani et al., 2018). Consequently, Gal-3 serves as a powerful biomarker for identifying high-risk AMI patients who warrant intensified monitoring and therapeutic strategies to mitigate the risk of HF development and death (Sygitowicz et al., 2021; Idzikowska et al., 2022; Hao et al., 2024).

### 5.2 Risk stratification: Galectin-3 for identifying high-risk AMI phenotypes

Gal-3 measurement significantly enhances the ability to stratify AMI patients into distinct risk categories, enabling more personalized management strategies. Elevated Gal-3 levels, particularly when measured early (within 72 h) post-AMI, effectively identify patients at substantially higher risk for adverse outcomes, independent of traditional risk factors (age, sex, comorbidities), clinical presentation, and standard biomarkers like troponin. Patients can be categorized into low, intermediate, and high-risk groups based on predefined Gal-3 thresholds (e.g., <17.8 ng/mL, 17.8–25.9 ng/mL, >25.9 ng/mL) or population-specific percentiles (e.g., median, 75th percentile) (Idzikowska et al., 2022; Winter et al., 2016; O'Donoghue et al., 2016). For instance, AMI patients with Gal-3 levels above the 75th percentile have a markedly increased risk of death, HF development, and HF hospitalization within the first year compared to those below the median (O'Donoghue et al., 2016; Kusaka et al., 2015). This stratification power extends to specific high-risk subgroups. Gal-3 levels are particularly prognostic in patients with diabetes mellitus, where they correlate strongly with underlying coronary atherosclerosis burden and plaque vulnerability (Ozturk et al., 2015), and in patients who develop atrial fibrillation (AF) post-AMI, where higher Gal-3 levels predict AF occurrence and are associated with worse outcomes in those with AF (Stanojevic et al., 2019) (Misirlioglu et al., 2025). Furthermore, Gal-3 aids in identifying patients with premature MI who remain at elevated long-term risk despite younger age (Winter et al., 2016). Its association with the severity of underlying coronary artery disease (CAD), even in chronic settings, provides context for its prognostic value in the acute event (Singsaas et al., 2016).

The true strength of Gal-3 for risk stratification lies in its incremental value when combined with other biomarkers and clinical parameters. Gal-3 provides complementary pathophysiological information reflecting fibrosis and chronic remodeling, distinct from markers of myocardial necrosis (troponin), hemodynamic stress (BNP/NT-proBNP), or acute inflammation (CRP). Integrating Gal-3 into multi-marker panels significantly improves risk prediction models beyond what is achievable with single markers or clinical scores alone. Combining Gal-3 with NT-proBNP and high-sensitivity troponin T (hs-TnT), for example, offers superior discrimination for predicting death or HF hospitalization post-AMI compared to any single marker or traditional risk factors (Idzikowska et al., 2022; O'Donoghue et al., 2016). The combination of Gal-3 with soluble suppression of tumorigenicity 2 (sST2), another fibrosis/remodeling biomarker, provides even greater predictive power for adverse LV remodeling and HF events (Gagno et al., 2019). A particularly promising approach involves measuring Gal-3 binding protein (Gal-3BP) alongside Gal-3. Gagno et al. (2019) demonstrated that the Gal-3/Gal-3BP ratio significantly improved risk stratification for death and HF after MI compared to Gal-3 alone, likely reflecting the functional activity of Gal-3. Recent studies also explore combining Gal-3 with novel markers like thrombomodulin or pentraxin-3, showing potential for enhanced prediction, especially in distinguishing STEMI from NSTEMI risk profiles (Misirlioglu et al., 2025). This multi-marker strategy, incorporating Gal-3, allows clinicians to more accurately pinpoint patients at the highest absolute risk who would derive the greatest benefit from intensive monitoring, early aggressive HF pharmacotherapy (e.g., sacubitril/valsartan, SGLT2 inhibitors), and close follow-up to prevent remodeling and HF progression.

### 5.3 Treatment monitoring: Galectin-3 as a dynamic indicator of therapeutic response

Serial measurement of circulating Gal-3 levels holds significant promise for monitoring treatment efficacy and disease activity following AMI. Successful and timely reperfusion therapy, particularly primary percutaneous coronary intervention (PCI), is associated with a subsequent decline in serum Gal-3 levels. Patients exhibiting a significant decrease in Gal-3 within the first week post-PCI often demonstrate better preservation of LVEF, reduced infarct size progression, and lower rates of adverse remodeling compared to those with persistently elevated or rising levels (Sharma et al., 2017; Ghorbani et al., 2018; Redondo et al., 2021). Pharmacological interventions also influence Gal-3 dynamics. Studies indicate that certain cardioprotective drugs can modulate Gal-3 expression or its downstream effects. For instance, high-intensity statin therapy (e.g., atorvastatin 80 mg or rosuvastatin 40 mg) initiated early post-AMI has been shown to significantly reduce serum Gal-3 levels over time compared to lower intensities or placebo, correlating with their pleiotropic anti-inflammatory and potentially anti-remodeling effects (Dong et al., 2018; Frangogiannis, 2023). Similarly, emerging evidence suggests that novel heart failure therapies like sodium-glucose cotransporter two inhibitors (SGLT2i) and sacubitril/valsartan may contribute to lowering Gal-3 levels, potentially reflecting their beneficial impact on cardiac fibrosis and inflammation. The trajectory of Gal-3 levels during follow-up provides valuable prognostic information; patients whose Gal-3 levels remain high or increase over time are at substantially greater risk for progressive ventricular dysfunction, heart failure hospitalization, and death, signaling the need for therapeutic intensification or closer monitoring (Ghorbani et al., 2018; Idzikowska et al., 2022). Furthermore, Gal-3 serves as a direct pharmacodynamic biomarker in trials of Gal-3 inhibitors (e.g., MCP, TD139 analogs). Effective target engagement by these inhibitors is often evidenced by a reduction in circulating Gal-3 levels or activity, which correlates with attenuation of inflammation, fibrosis, and improved functional outcomes in preclinical models, paving the way for its use in monitoring response in future clinical trials (Wang et al., 2023; Ibarrola et al., 2019). Thus, tracking Gal-3 trends offers a non-invasive window into the underlying biological response to therapy, aiding clinicians in personalizing post-AMI management.

## 6 Intervention strategies: targeting Galectin-3 in AMI

### 6.1 Genetic and molecular interventions

Preclinical studies robustly demonstrate that genetic ablation of Gal-3 confers significant cardioprotection post-AMI. *Lgals3* ^−/−^ mice exhibit markedly attenuated inflammation (reduced macrophage infiltration and TNF-α/IL-6 levels), suppressed fibrosis (decreased collagen deposition and myofibroblast activation), diminished cardiomyocyte apoptosis, and improved left ventricular function compared to wild-type controls (Cassaglia et al., 2020; Martínez-Martínez et al., 2015). Building on this, novel delivery systems aim for targeted Gal-3 knockdown. Ultrasound-guided microbubble cavitation effectively delivers Gal-3-specific shRNA to the infarcted myocardium, silencing Gal-3 expression locally. This approach significantly reduces fibrosis and improves cardiac output in rodent AMI models, offering spatial precision and reduced off-target effects (Li et al., 2023). Additionally, endogenous inhibitory peptides (e.g., those disrupting Gal-3 oligomerization) show promise in mitigating adverse remodeling in models of cardiac-specific Gal-3 overexpression, providing an alternative molecular strategy (Sonkawade et al., 2021) (Table 2).

**TABLE 2:** Pharmacological and genetic interventions targeting Galectin-3 in AMI.

Intervention	Type	Mechanism	Model/Stage	Key effects
MCP	Natural inhibitor	Competes with CRD binding	Rodent/Pig AMI	Reduces fibrosis, inflammation, apoptosis
TD139/GB0139	Small molecule	High-affinity CRD binding	Preclinical/Phase II (IPF)	Attenuates remodeling, improves function
Gal-3 shRNA	Genetic	siRNA-mediated knockdown	Rodent AMI	Reduces fibrosis, improves output
Statins	Repurposed drug	Pleiotropic downregulation of Gal-3	Clinical AMI	Lowers Gal-3, improves outcomes

Abbreviations: Gal-3, Galectin-3; MCP, modified citrus pectin; CRD, carbohydrate recognition domain; AMI, acute myocardial infarction.

### 6.2 Pharmacological inhibition

Pharmacological blockade of Gal-3 is a rapidly advancing therapeutic Frontier (Table 2):

MCP: A natural polysaccharide derivative, MCP competitively binds Gal-3’s CRD. Administered post-AMI in rodents and pigs, MCP reduces cardiac Gal-3 activity, dampens NF-κB-driven inflammation and TGF-β/Smad3 signaling, attenuates oxidative/ER stress, decreases infarct size, and improves systolic function (Lu et al., 2017; Wang et al., 2023; Ibarrola et al., 2019). Its safety profile supports clinical exploration.

High-Affinity Small Molecule Inhibitors: Synthetic compounds offer greater specificity and potency. TD139 and next-generation glycomimetics (e.g., GB0139, GB1211) bind the Gal-3 CRD with nanomolar affinity. These inhibitors significantly reduce fibrosis, inflammation, and apoptosis in cardiac I/R and remodeling models (Mo et al., 2019; Wang et al., 2023; Ibarrola et al., 2019). Some are in clinical trials for pulmonary fibrosis (GB0139), paving the path for cardiovascular applications.

Repurposed Drugs: Certain existing drugs (e.g., high-intensity statins) reduce circulating Gal-3 levels post-AMI, potentially contributing to their pleiotropic benefits (Tunçez et al., 2019). SGLT2 inhibitors and sacubitril/valsartan are being investigated for Gal-3 modulation.

Proof-of-concept studies confirm that pharmacological Gal-3 inhibition, particularly when initiated early post-MI or during I/R, effectively blunts key pathological pathways, improving survival and functional outcomes (Mosleh et al., 2018; Frangogiannis, 2023).

## 7 Controversies and challenges in clinical translation

### 7.1 Variability in detection: methodology, timing, and standard thresholds

A primary barrier to clinical implementation is the lack of standardization in Gal-3 detection. Significant inter-assay variability across different commercial platforms compromises results comparability and hampers the establishment of universal diagnostic cut-offs (Asleh et al., 2019; Ghorbani et al., 2018; Dong et al., 2018). Its dynamic post-AMI expression profile further complicates the determination of the optimal sampling time for reliable risk prediction (Sharma et al., 2017; Meijers et al., 2015). The absence of consensus on clinically validated, pathology-specific thresholds (e.g., for STEMI vs. NSTEMI) prevents its consistent integration into decision-making pathways (Kokturk et al., 2023; Idzikowska et al., 2022).

### 7.2 Pathophysiological specificity: influence of comorbidities and context-dependent roles

The interpretation of circulating Gal-3 is confounded by its limited organ specificity. Levels are elevated by common comorbidities in AMI patients, such as chronic kidney disease, atrial fibrillation, and diabetes, which obscures its specific link to post-infarct remodeling (Kovacevic et al., 2018; Ozturk et al., 2015; Stanojevic et al., 2019). Furthermore, Gal-3 exhibits functional duality; while it drives maladaptive processes, evidence suggests it may also participate in early reparative responses, such as debris clearance (Prabhu and Frangogiannis, 2016; Frangogiannis, 2023). This context-dependency, highlighted by increased susceptibility to myocarditis in knockout models, raises concerns about the safety of systemic inhibition (Kovacevic et al., 2018).

### 7.3 Challenges in targeted therapy: drug development, delivery, and personalized medicine

Translating Gal-3 inhibition into therapy faces practical hurdles. Drug delivery is challenging due to Gal-3’s intracellular and extracellular actions. Current inhibitors like MCP have limited bioavailability and cardiac specificity (Arias et al., 2014; De Leo et al., 2020). Advanced strategies (e.g., microbubble-assisted gene delivery) remain preclinical (Li et al., 2023; De Leo et al., 2020). Clinical trial design is complicated by the need for precise patient stratification using a non-standardized biomarker and by demonstrating incremental benefit over existing therapies. Additionally, individual variability in Gal-3 biology necessitates a personalized medicine approach to identify which patients will benefit most from inhibition (Frangogiannis, 2023).

## 8 Conclusion

In conclusion, Galectin-3 emerges as a master regulator of post-infarction pathophysiology, orchestrating adverse remodeling through interconnected mechanisms of inflammation, fibrosis, apoptosis, and oxidative/ER stress. Its dynamic expression profile underpins its strong utility as a prognostic biomarker for predicting heart failure and mortality, enhancing risk stratification beyond conventional markers. Preclinical studies robustly validate Gal-3 inhibition as a promising therapeutic strategy, effectively attenuating cardiac dysfunction. However, clinical translation necessitates overcoming challenges related to its context-dependent biology, optimal therapeutic window, and drug delivery. Future efforts must focus on biomarker-guided, comorbidity-stratified trials to fully realize the potential of targeting Gal-3 and improving long-term outcomes after AMI.
